# Unraveling the metabolic underpinnings of frailty using multicohort observational and Mendelian randomization analyses

**DOI:** 10.1111/acel.13868

**Published:** 2023-05-15

**Authors:** Jonathan K. L. Mak, Laura Kananen, Chenxi Qin, Ralf Kuja‐Halkola, Bowen Tang, Jake Lin, Yunzhang Wang, Tuija Jääskeläinen, Seppo Koskinen, Yi Lu, Patrik K. E. Magnusson, Sara Hägg, Juulia Jylhävä

**Affiliations:** ^1^ Department of Medical Epidemiology and Biostatistics Karolinska Institutet Stockholm Sweden; ^2^ Faculty of Social Sciences (Health Sciences) and Gerontology Research Center (GEREC) University of Tampere Tampere Finland; ^3^ Institute for Molecular Medicine Finland FIMM, Helsinki Institute of Life Science HiLIFE, University of Helsinki Helsinki Finland; ^4^ Department of Clinical Sciences, Danderyd Hospital Karolinska Institutet Stockholm Sweden; ^5^ Finnish Institute for Health and Welfare Helsinki Finland; ^6^ Department of Global Public Health Karolinska Institutet Stockholm Sweden

**Keywords:** biomarkers, frailty, Mendelian randomization, metabolomics, twins

## Abstract

Identifying metabolic biomarkers of frailty, an age‐related state of physiological decline, is important for understanding its metabolic underpinnings and developing preventive strategies. Here, we systematically examined 168 nuclear magnetic resonance‐based metabolomic biomarkers and 32 clinical biomarkers for their associations with frailty. In up to 90,573 UK Biobank participants, we identified 59 biomarkers robustly and independently associated with the frailty index (FI). Of these, 34 associations were replicated in the Swedish TwinGene study (*n* = 11,025) and the Finnish Health 2000 Survey (*n* = 6073). Using two‐sample Mendelian randomization, we showed that the genetically predicted level of glycoprotein acetyls, an inflammatory marker, was statistically significantly associated with an increased FI (*β* per SD increase = 0.37%, 95% confidence interval: 0.12–0.61). Creatinine and several lipoprotein lipids were also associated with increased FI, yet their effects were mostly driven by kidney and cardiometabolic diseases, respectively. Our findings provide new insights into the causal effects of metabolites on frailty and highlight the role of chronic inflammation underlying frailty development.

## Graphical abstract

This study explored the relationships between frailty and circulating metabolic biomarkers measured using a standardized nuclear magnetic resonance (NMR) metabolomics platform in three large European population‐based studies. Out of the 200 assessed biomarkers, 34 were found to be robustly associated with frailty in the observational analysis. Two‐sample Mendelian randomization further implicated potential causal relationships of glycoprotein acetyls, creatinine, and several lipoprotein lipids with frailty.

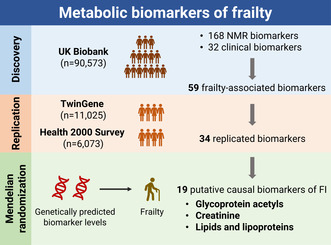

**Abbreviations**
BMI: body mass index
CI: confidence interval
CRP: C‐reactive protein
DZ: dizygotic
FDR: false discovery rate
FI: frailty index
FP: frailty phenotype
GlycA: glycoprotein acetyls
GWAS: genome‐wide association study
HbA1c: glycated hemoglobin
IV: instrumental variable
IVW: inverse variance weighted
LASSO: least absolute shrinkage and selection operator
LDL: low‐density lipoprotein
MR: Mendelian randomization
MR‐PRESSO: MR‐pleiotropy residual sum and outlier
MZ: monozygotic
NMR: nuclear magnetic resonance
SALT: Screening Across the Lifespan Twin Study
SD: standard deviation
SNP: single nucleotide polymorphism
UKB: UK Biobank

## 1 INTRODUCTION

Frailty, a state of multisystem physiological decline (Clegg et al., 2013), is a strong, independent predictor of a range of adverse outcomes, such as mortality, falls, and hospitalizations (Kojima, 2015, 2016; Peng et al., 2022). Among the multiple operational definitions of frailty, the two most widely adopted models are the frailty index (FI) (Searle et al., 2008) and the frailty phenotype (FP) (Fried et al., 2001). The FI describes frailty as the accumulation of age‐related health deficits (e.g., diseases, signs, symptoms, and disabilities), whereas the FP considers frailty as a clinical syndrome characterized by weakness, slowness, exhaustion, unintentional weight loss, and low physical activity. Depending on the assessment method, the overall prevalence of frailty varies from 12% to 24% among individuals aged ≥50 years and it rises substantially with age (O'Caoimh et al., 2021). Since frailty is dynamic and potentially reversible (Hoogendijk et al., 2019), improved diagnosis and management of frail individuals is crucial to reduce morbidity and mortality in the aging population.

Due to its complexity and multidimensional nature, it is challenging to uncover the underlying mechanisms of frailty. Studies have shown that both genetic and environmental factors play an important role in the etiology of frailty, with an estimated heritability of 25%–50% (Livshits et al., 2018; Mak et al., 2021). A recent genome‐wide association study (GWAS) provided further insights into the genetic underpinnings of frailty, suggesting that frailty is influenced by genetic loci related to several disease risk factors, such as body mass index (BMI), cardiovascular diseases, and mental health (Atkins et al., 2021). However, how these genetic findings translate into the biological processes underlying frailty is still unclear. Studying metabolic biomarkers, which are small molecules involved in metabolic reactions and regulated by genotypes to a varying degree, could contribute to the understanding of the molecular mechanisms of frailty and aid in the development of preventive strategies (Picca et al., 2019). Prior studies have proposed a plethora of frailty‐associated blood biomarkers, including inflammation markers (e.g., C‐reactive protein [CRP], interleukin‐6), immune markers (e.g., white blood cell count), hormones (e.g., testosterone, insulin‐like growth factor 1), and clinical markers (e.g., albumin, creatinine) (Cardoso et al., 2018; Kane & Sinclair, 2019; Picca et al., 2022). More recently, metabolomics studies based on liquid chromatography–mass spectrometry suggested that metabolites involving in energy producing pathways and antioxidation could be associated with frailty (Kameda et al., 2020; Rattray et al., 2019; Westbrook et al., 2021). However, no biomarker has been identified so far that could be used as a specific target for frailty diagnosis and drug development. One of the reasons is that current evidence is mostly based on observational studies, which is difficult to establish causal relationships as the findings may be biased by confounding and reverse causality.

As a causal inference method, Mendelian randomization (MR) uses genetic variants as instrumental variables (IVs) to study the lifelong effect of an exposure on a disease outcome, providing an approach that is less prone to confounding and reverse causation compared to observational studies (Davies et al., 2018). To date, a few MR studies have identified causal links between increased low‐density lipoprotein (LDL)‐cholesterol, saturated fatty acids, as well as decreased serum total protein levels, and the FI (Tomata et al., 2021, 2022; Wang et al., 2019). Nevertheless, whether other frailty‐associated metabolic biomarkers may also have causal effects on frailty, and whether there are differences in the metabolic underpinnings between the different constructs of frailty (e.g., FI vs. FP) remain largely unexplored.

**FIGURE 1:**
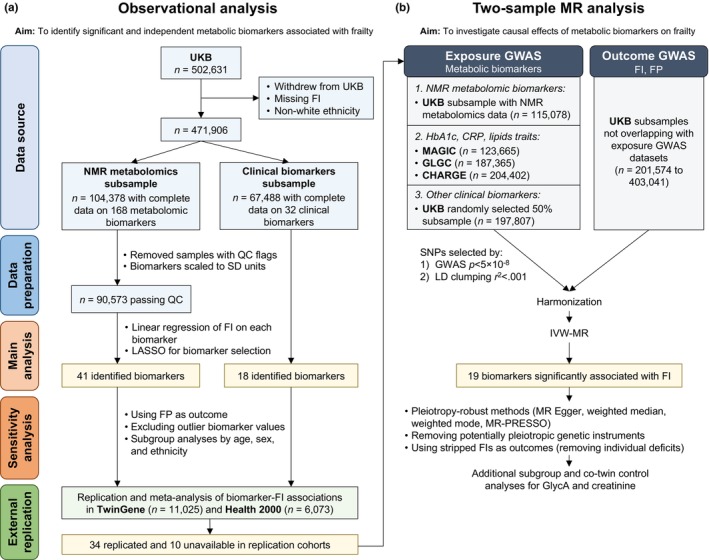
Study overview. This study was split into two parts: observational and MR analysis. (a) In observational analysis, 90,573 and 67,488 white UKB participants who had complete data on 168 NMR metabolomic and 32 clinical biomarkers, respectively, were used to assess the cross‐sectional associations between the biomarkers and the frailty index. A total of 41 metabolomic and 18 clinical biomarkers that were statistically significant in linear regression models after Bonferroni correction (*p* < 0.05/200) and had nonzero coefficients in LASSO models were brought forward to the replication phase in TwinGene and Health 2000. (b) Two‐sample MR analyses were performed for the 34 replicated biomarkers (and 10 biomarkers unavailable in the replication cohorts). CHARGE, Cohorts for Heart and Aging Research in Genomic Epidemiology consortium; CRP, C‐reactive protein; FI, frailty index; FP, frailty phenotype; GLGC, Global Lipids Genetics Consortium; GWAS, genome‐wide association study; HbA1c, glycated hemoglobin; LASSO, least absolute shrinkage and selection operator; LD, linkage disequilibrium; MAGIC, Meta‐Analyses of Glucose and Insulin‐related traits Consortium; MR, Mendelian randomization; MR‐PRESSO, MR‐pleiotropy residual sum and outlier; NMR, nuclear magnetic resonance; QC, quality control; SD, standard deviation; SNP, single nucleotide polymorphism; UKB, UK Biobank.

To address these knowledge gaps and identify novel metabolic biomarkers of frailty, we investigated the effects of 200 circulating metabolic biomarkers on frailty, measured by both the FI and FP, using observational and MR approaches (Figure 1). The analyzed biomarkers include 168 metabolomic biomarkers quantified from a standardized, high‐throughput nuclear magnetic resonance (NMR) metabolomics platform, as well as 32 conventional clinical biomarkers from serum and urine samples. Using data from three European population‐based studies, including the UK Biobank (UKB) as discovery cohort, and the Swedish TwinGene study and the Finnish Health 2000 Survey as replication cohorts, we identified 34 biomarkers consistently and strongly associated with frailty independent of other risk factors. Subsequently, we conducted two‐sample MR analyses to examine whether the identified biomarkers are causally related to frailty.

## 2 RESULTS

### 2.1 Cross‐sectional associations of metabolic biomarkers with frailty in UK biobank

Details of the 200 metabolic biomarkers are shown in Table S1 and Figures S1 and S2. The 168 NMR metabolomic biomarkers include amino acids, cholesterols, lipoproteins, fatty acids, and metabolites related to inflammation and fluid balance; part of the metabolites also overlaps with the included clinical biomarkers, such as LDL‐cholesterol, creatinine, and albumin. As expected, high correlations were found across many of these biomarkers, especially those within the same biological domains (Figures S3 and S4; Tables S2 and S3). Frailty was assessed using the FI (ranging from 0% to 100%) and FP scores (ranging from 0 to 5, as a secondary outcome), where higher scores denote higher degrees of frailty (Tables S4 and S5). Two subsamples from the UKB were used as the discovery cohorts for the metabolomic and clinical biomarkers, respectively. They consisted of 90,573 participants who had complete data on the 168 NMR metabolomic biomarkers (mean age 56.8 years [standard deviation (SD) 8.0]; 54% women; mean FI 12.3% [SD 7.4]) and 67,488 participants who had complete data on the 32 clinical biomarkers (mean age 57.5 years [SD 8.2]; 39% women; mean FI 13.0% [SD 7.7]; Table 1).

**TABLE 1:** Characteristics of the samples used in the observational analysis.

Characteristic	UK biobank (discovery)^a^		TwinGene (replication)	Health 2000 (replication)
	NMR metabolomics subsample	Clinical biomarkers subsample		
No. of participants	90,573	67,488	11,025	6073
Age at baseline, year				
Mean ± SD	56.77 ± 8.03	57.46 ± 8.16	58.33 ± 7.91	52.54 ± 14.68
Range	40–71	39–72	41–87	30–97
Women, *n* (%)	49,296 (54.4)	26,379 (39.1)	6017 (54.6)	3325 (54.8)
BMI, kg/m^2^, mean ± SD	27.33 ± 4.69	28.42 ± 5.16	25.05 ± 3.34	26.92 ± 4.63
Current smokers, *n* (%)	8964 (9.9)	7751 (11.5)	1795 (16.3)	1305 (21.5)
Alcohol consumption, g/year, mean ± SD	–	–	–	3599 ± 8220
Less than weekly, *n* (%)	25,790 (28.5)	19,321 (28.6)	3122 (30.1)	–
Weekly, *n* (%)	64,727 (71.5)	48,134 (71.4)	7241 (69.9)	–
Education level^b^, *n* (%)				
High	29,213 (32.5)	20,223 (30.3)	2862 (26.0)	1754 (28.9)
Intermediate	44,855 (50.0)	33,384 (50.0)	5199 (47.2)	1983 (32.7)
Low	15,684 (17.5)	13,206 (19.8)	2951 (26.8)	2336 (38.5)
Deprivation index^c^, mean ± SD	−1.48 ± 3.00	−1.25 ± 3.10	–	–
FI^d^, %, mean ± SD	12.29 ± 7.41	13.03 ± 7.71	12.15 ± 8.04	17.67 ± 12.90
FP score^e^, mean ± SD	0.56 ± 0.82	0.64 ± 0.87	–	–

^a^ Two subsamples from the UK Biobank cohort were used for analysis of the two groups of biomarkers. The NMR metabolomics subsample had complete data on the 168 metabolomic biomarkers, while the clinical biomarkers subsample had complete data on the 32 clinical biomarkers.

^b^ Education level in UKB was assessed by the highest self‐reported qualification: low (no relevant qualifications); intermediate (A levels, O levels/GCSEs, CSEs, NVQ/HND/HNC, and other professional qualifications); high (college or university degree). Education level in TwinGene was defined by years of completed education: low (<9 years); intermediate (9–12 years); high (>12 years).

^c^ Townsend deprivation index was derived from national census data regarding unemployment, car ownership, home ownership, and household overcrowding. A higher score indicates a higher level of socioeconomic deprivation. It was only available in UK Biobank.

^d^ FI was multiplied by 100 and was considered as the percentage of deficit accumulation (from 0% to 100%).

^e^ FP was considered as a continuous score representing the number of frailty criteria present (from 0 to 5). It was only available in UK Biobank.

Abbreviations: BMI, body mass index; FI, frailty index; FP, frailty phenotype; NMR, nuclear magnetic resonance; SD, standard deviation.

**FIGURE 2:**
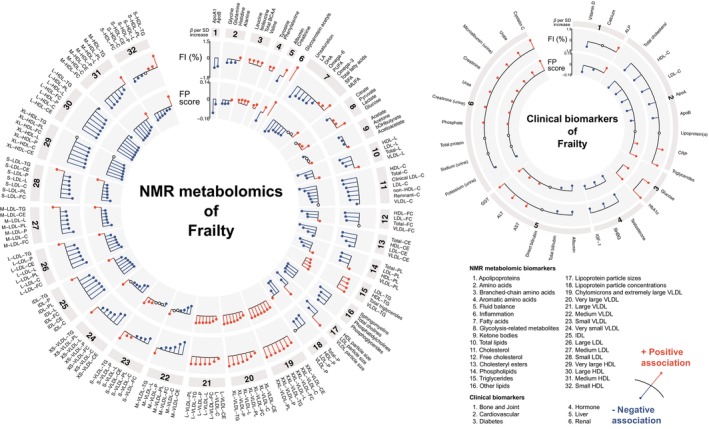
Age‐ and sex‐adjusted observational associations of metabolic biomarkers with FI and FP scores in the UK Biobank. Estimates represent the age‐ and sex‐adjusted changes in frailty index (outer tracks of the circles) or frailty phenotype score (inner tracks of the circles) per 1 standard deviation increase in the biomarker level. Red dots indicate positive associations, whereas blue dots indicate negative associations. Filled dots represent statistically significant associations after Bonferroni correction at *p* < 0.00025 (i.e., 0.05/200, considering 200 biomarkers). The corresponding numeric estimates are shown in Tables S6 and S7. ALP, alkaline phosphatase; ALT, alanine aminotransferase; AST, aspartate aminotransferase; ApoA, apolipoprotein A; ApoB, apolipoprotein B; BCAA, branched chain amino acids; bOHbutyrate, 3‐hydroxybutyrate; C, cholesterol; CE, cholesteryl esters; DHA, docosahexaenoic acid; FC, free cholesterol; FI, frailty index; FP, frailty phenotype; GGT, gamma glutamyltransferase; HbA1c, glycated hemoglobin; HDL, high‐density lipoproteins; IDL, intermediate‐density lipoproteins; IGF, insulin‐like growth factor; L, large (when used as prefix) or total lipids (when used as suffix); LA, linoleic acid; LDL, low‐density lipoproteins; M, medium; MUFA, monounsaturated fatty acids; NMR, nuclear magnetic resonance; P, particle concentrations; PL, phospholipids; PUFA, polyunsaturated fatty acids; S, small; SD, standard deviation; SFA, saturated fatty acids; SHBG, sex hormone‐binding globulin; TG, triglycerides; Unsaturation, degree of unsaturation; UKB, UK Biobank; VLDL, very‐low‐density lipoproteins; XL, very large; XS, very small; XXL, extremely large.

Using linear regression models adjusted for age and sex, we found 191 of the 200 metabolic biomarkers statistically significantly associated with the FI after Bonferroni correction for multiple testing at *p* < 0.00025 (i.e., 0.05/200; Figure 2; Tables S6 and S7). Among the NMR metabolomic biomarkers, glycoprotein acetyls (GlycA) had the strongest positive association, every SD increase (equivalent to 0.11 mmol/L) being associated with a 1.40% higher FI (95% confidence interval [CI]: 1.35–1.44). Most of the cholesterols and lipoproteins were negatively associated with the FI (Figure 2). Among the clinical biomarkers, the largest effect sizes were found for glycated hemoglobin (HbA1c; *β* per SD increase: 1.84%, 95% CI: 1.78–1.90) and total cholesterol (*β* per SD increase: −1.62%, 95% CI: −1.67 to −1.56). When additionally adjusted for baseline assessment center, BMI, smoking, alcohol consumption, education, and deprivation index (i.e., fully adjusted models), we found that 164 biomarkers remained statistically significantly, where most of them were inversely, associated with the FI (Figure S5). As the metabolic biomarkers were highly intercorrelated, we applied the least absolute shrinkage and selection operator (LASSO) procedure in the observational analysis to select biomarkers that were strongly and independently associated with the FI when adjusted for each other and also for age and sex. In total, 56 NMR metabolomic and 21 clinical biomarkers were identified in LASSO models (i.e., had nonzero coefficients; Figure S6; Tables S6 and S7).

Several sensitivity analyses were performed. Firstly, instead of the FI, we used FP score as the outcome and found that most of the associations were directionally consistent with the analyses using the FI (Figure 2 and Figure S5). Secondly, after excluding outlier biomarker values, all the biomarker‐FI associations remained essentially unchanged (Tables S6 and S7). Finally, we performed stratified analyses and observed largely similar results in subgroups by age, sex, and in non‐white ethnic groups (Figure S7; Tables S8 and S9).

### 2.2 Replication in TwinGene and health 2000

**FIGURE 3:**
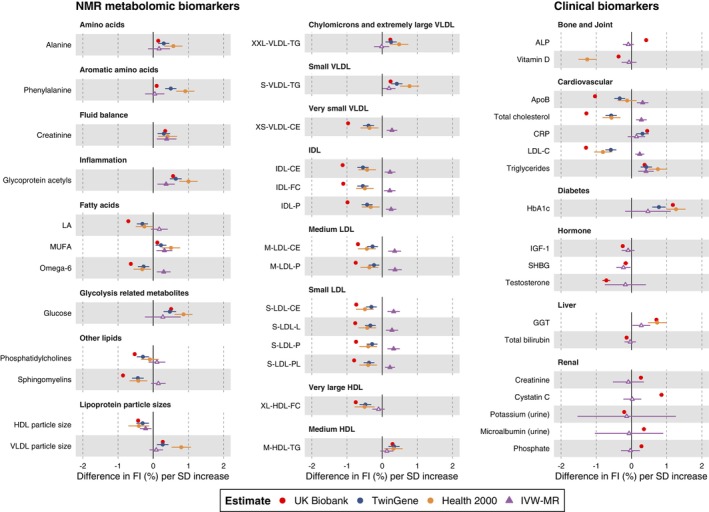
Observational and MR effect estimates of selected metabolic biomarkers on FI. The 44 included biomarkers were those identified as FI‐associated biomarkers in the UK Biobank (*p* < 0.00025 in linear regression models and had nonzero coefficients in LASSO models) and replicated (or not available) in TwinGene and Health 2000. The estimates are from fully adjusted linear regression models, including age, sex, baseline assessment center (only in UK Biobank), body mass index, smoking, alcohol consumption, education level, and deprivation index (only in UK Biobank) as covariates. The models in TwinGene were additionally corrected for twin relatedness. For the IVW‐MR estimates, filled triangles represent statistically significant associations at *p* < 0.011 (FDR‐corrected *p* value threshold). All the effect sizes represent the changes in FI (%) per 1 standard deviation increase in biomarker level (except for the IVW‐MR estimates for CRP and HbA1c, which are per log mg/L increase and per % increase, respectively; details of the units used are shown in Table S11). All the observational estimates are shown in Tables S6 and S7; MR estimates are shown in Table S14. ALP, alkaline phosphatase; ApoB, apolipoprotein B; C, cholesterol; CE, cholesteryl esters; FC, free cholesterol; FI, frailty index; GGT, gamma glutamyltransferase; HbA1c, glycated hemoglobin; HDL, high‐density lipoproteins; IDL, intermediate‐density lipoproteins; IGF, insulin‐like growth factor; IVW, inverse variance weighted; L, total lipids; LA, linoleic acid; LDL, low‐density lipoproteins; M, medium; MR, Mendelian randomization; MUFA, monounsaturated fatty acids; NMR, nuclear magnetic resonance; P, particle concentrations; PL, phospholipids; PUFA, polyunsaturated fatty acids; S, small; SD, standard deviation; SHBG, sex hormone‐binding globulin; TG, triglycerides; Unsaturation, degree of unsaturation; VLDL, very‐low‐density lipoproteins; XL, very large; XS, very small; XXL, extremely large.

For 41 NMR metabolomic biomarkers and 18 clinical biomarkers that were (i) significantly associated with the FI in multivariable‐adjusted models and (ii) selected by LASSO in the UKB, we further examined their associations with the FI in two independent samples, including 11,025 Swedish TwinGene participants (mean age 58.3 years [SD 7.9]; 55% women; mean FI 12.1% [SD 8.0]) and 6073 Finnish Health 2000 participants (mean age 52.5 years [SD 14.7]; 55% women; mean FI 17.7% [SD 12.9]; Table 1). We meta‐analyzed the biomarker‐FI associations in TwinGene and Health 2000 and found that out of the 49 biomarkers that were available in the replication cohorts, 34 were significantly associated with the FI (*p* < 0.05; Table S10). The replicated biomarkers were NMR metabolomic biomarkers from several domains including amino acids (e.g., alanine, phenylalanine), fluid balance (e.g., creatinine), inflammation (GlycA), fatty acids (e.g., monounsaturated fatty acids, linoleic acid), and lipoprotein subclasses, as well as clinical biomarkers such as LDL‐cholesterol, CRP, and HbA1c (Figure 3).

### 2.3 Two‐sample Mendelian randomization of identified biomarkers on frailty

**FIGURE 4:**
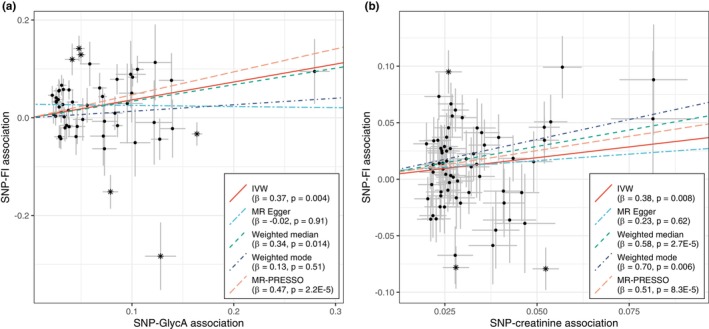
MR scatter plots for the effects of NMR‐derived glycoprotein acetyls and creatinine on FI. (a) Scatter plot of the SNP‐FI associations against SNP‐GlycA associations. The slopes of the colored lines represent the estimated change in FI (%) per 1 standard deviation increase in genetically predicted GlycA level. Intercepts for IVW, weighted median, weighted mode, and MR‐PRESSO were fixed at 0; the MR‐Egger intercept was 0.0276 (*p* = 0.028), indicating potential directional pleiotropy. Fifty‐five SNPs were used in total, six of which were identified as outliers by MR‐PRESSO (rs10455872, rs117733303, rs1548306, rs72801474, rs77303550, and rs9270074; these SNPs were indicated by asterisks). (b) Scatter plot of the SNP‐FI associations against the SNP‐creatinine associations. The slopes of the colored lines represent the estimated change in FI (%) per 1 standard deviation increase in genetically predicted creatinine level. The MR‐Egger intercept was 0.005 (*p* = 0.73), indicating no evidence of directional pleiotropy. Sixty‐nine SNPs were used in total, three of which were identified as outliers by MR‐PRESSO (rs10008637, rs3974479, and rs9272116); these SNPs were indicated by asterisks. Error bars represent 95% confidence intervals. All the MR estimates are shown in Table S14. FI, frailty index; GlycA, glycoprotein acetyls; IVW, inverse variance weighted; MR, Mendelian randomization; MR‐PRESSO, MR‐pleiotropy residual sum and outlier; SNP, single nucleotide polymorphism.

Next, we performed two‐sample MR analyses to examine potential causal relationships of the 44 biomarkers, of which 34 were replicated in TwinGene and Health 2000 and 10 were unavailable in the replication cohorts. Genetic instruments (i.e., single nucleotide polymorphisms [SNPs] associated with the biomarkers) were selected from the largest available GWASs; the estimated *F*‐statistics for all instruments were > 10 (Tables S11–S13). To obtain summary statistics for the SNP‐frailty (outcome) associations, we performed a GWAS for the FI and FP in UKB samples that did not have an overlap of individuals with the exposure GWASs. Using the inverse variance weighted (IVW)‐MR method, we observed 18 significant associations with the FI at a false discovery rate (FDR)‐corrected threshold of *p* < 0.011 (Figure 3 and Table S14). Several of these MR estimates were directionally consistent with the observational estimates. For instance, each SD increment in the genetically predicted levels of GlycA and creatinine were associated with 0.37% (95% CI: 0.12–0.61) and 0.38% (95% CI: 0.10–0.66) increase in the FI, respectively (Figure 4). By contrast, omega‐6, apolipoprotein B, total cholesterol, LDL‐cholesterol, and some of the lipoprotein subclasses were associated with the FI negatively in the observational analysis but positively in the IVW‐MR (Figure 3). None of the selected 44 biomarkers were statistically significantly associated with FP score (Table S14).

Notably, a considerable heterogeneity was observed for most MR estimates (*p* < 0.05 from Cochran's *Q* tests; Table S14), some of which could possibly be due to horizontal pleiotropy (i.e., genetic variants associate with other traits that influence the outcome). As sensitivity analyses, we applied pleiotropy‐robust methods including MR‐Egger, weighted median, weighted mode, and MR‐pleiotropy residual sum and outlier (MR‐PRESSO). Estimates for most biomarkers were comparable when using different MR methods; however, we found evidence of directional pleiotropy for GlycA, monounsaturated fatty acids, and total lipids in small LDL (*p* < 0.05 for MR‐Egger intercept; Table S14).

Since many of the IVs were associated with >1 NMR metabolomic biomarkers, we performed a sensitivity analysis by excluding the potentially pleiotropic SNPs from each biomarker. As shown in Table S15, the MR estimates for GlycA and creatinine remained robust, and we also observed a statistically significant association between genetically predicted GlycA and increased FP score (*β* per SD increase: 0.040, 95% CI: 0.012–0.067). However, the MR estimates for most of the lipids and lipoproteins were attenuated, probably due to the highly reduced number of genetic instruments.

**FIGURE 5:**
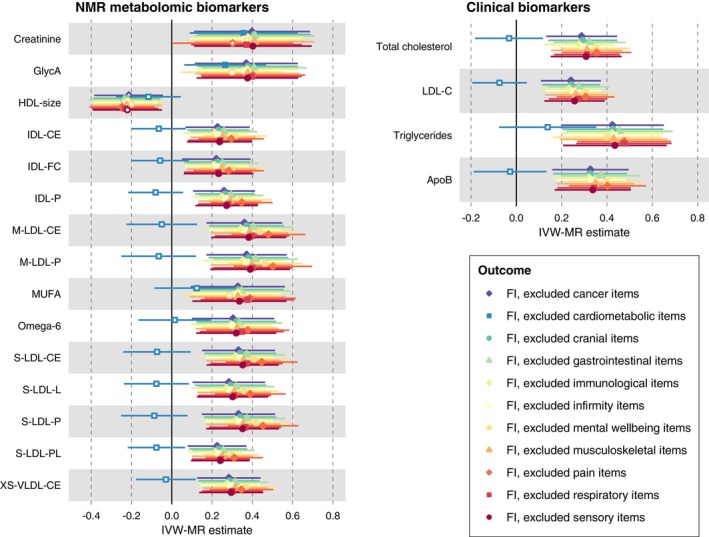
MR sensitivity analysis for the effects of metabolic biomarkers on 11 stripped FIs removing deficit items from each category. The 19 included biomarkers are those that were significantly associated with the FI in IVW‐MR after FDR correction. The 11 stripped FIs were calculated as the sum of deficit items divided by the total, after excluding items from each category in the UK Biobank. For example, the “FI, excluded cardiometabolic items” was a 41‐item FI removing 8 cardiometabolic items (i.e., diabetes, myocardial infarction, angina, stroke, high blood pressure, hypothyroidism, deep vein thrombosis, and high cholesterol). The list of FI items in each category is shown in Table S4. A GWAS and a two‐sample MR analysis were then performed for each of the 11 stripped FIs, using the same method as in the main analysis. The IVW‐MR effect sizes represent the difference in FI (%) per 1 SD increase in genetically predicted biomarker level. Filled symbols represent statistically significant associations at *p* < 0.011 (FDR‐corrected *p* value threshold). The numeric point estimates from this sensitivity analysis are shown in Table S16. ApoB, apolipoprotein B; C, cholesterol; CE, cholesteryl esters; FC, free cholesterol; FI, frailty index; GlycA, glycoprotein acetyls; HDL, high‐density lipoproteins; IDL, intermediate‐density lipoproteins; IVW, inverse variance weighted; L, total lipids; LDL, low‐density lipoproteins; M, medium; MR, Mendelian randomization; MUFA, monounsaturated fatty acids; NMR, nuclear magnetic resonance; P, particle concentrations; PL, phospholipids; S, small; VLDL, very low‐density lipoproteins; XL, very large; XS, very small.

To further examine whether the effects of the biomarkers on the FI could be influenced by the deficit items included in the FI, we performed sensitivity analyses of the MR using 11 stripped FIs as outcomes, where deficit items from each of the 11 categories, such as cardiometabolic, cancer, and immunological items were removed from the corresponding FI. When removing cardiometabolic items (e.g., heart failure, stroke, diabetes, and high blood pressure), the IVW‐MR estimates for monounsaturated fatty acids, omega‐6, cholesterols, and lipoprotein subclasses were attenuated to null (Figure 5 and Table S16). Estimates for GlycA and creatinine remained significant across all the stripped FIs.

### 2.4 Subgroup and co‐twin control analyses of creatinine and GlycA on FI

**FIGURE 6:**
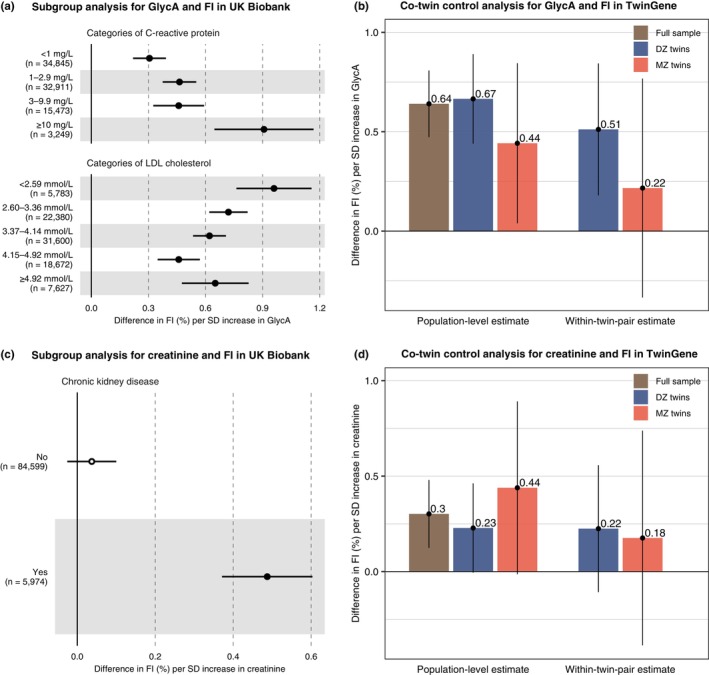
Subgroup and co‐twin control analyses for the association of NMR‐derived glycoprotein acetyls and creatinine with FI. (a) Forest plot showing the association between GlycA and FI across subgroups of C‐reactive protein and LDL‐cholesterol levels in the UK Biobank. LDL‐cholesterol values were adjusted for statin use. Details of the estimates are shown in Table S17. (b) Bar graph showing the population‐level and within‐twin‐pair estimates for the association between GlycA and FI in the full sample, DZ twins (2762 pairs), and MZ twins (1132 pairs) in TwinGene. (c) Forest plot showing the association between the NMR‐derived creatinine and FI among individuals with and without a chronic kidney disease. Chronic kidney disease cases were defined based on a self‐reported kidney disease at baseline and ICD‐10 codes N17, N18, or N19 from hospital data. Open circle represents a nonsignificant estimate (*p* ≥ 0.00025). Details of the estimates are shown in Table S18. (d) Bar graph showing the population‐level and within‐twin‐pair estimates for the association between the NMR‐derived creatinine and FI in the full sample, DZ twins (2762 pairs), and MZ twins (1132 pairs) in TwinGene. Models for the subgroup analysis were multivariable linear regression models adjusted for age, sex, baseline assessment center, body mass index, smoking, alcohol, education, and deprivation. Models for the co‐twin control analysis were generalized estimating equations adjusted for age, sex, body mass index, smoking, alcohol consumption, and years of education. All the error bars represent 95% confidence intervals. DZ, dizygotic; FI, frailty index; GlycA, glycoprotein acetyls; LDL, low‐density lipoprotein; NMR, nuclear magnetic resonance; SD, standard deviation.

For GlycA and creatinine that had putative causal relationships with the FI, we performed additional subgroup analyses in the UKB to assess whether the associations could be driven by their associated traits, namely CRP and LDL‐cholesterol for GlycA (Connelly et al., 2017), and chronic kidney disease for creatinine (Levey et al., 2009). The GlycA‐FI association was robust across all subgroups (individuals stratified by their CRP and LDL‐cholesterol levels), though it tended to be stronger among participants with high CRP and low LDL‐cholesterol levels (Figure 6a and Table S17). The creatinine‐FI association was statistically significant only in participants with a chronic kidney disease (Figure 6c and Table S18).

As a triangulation approach, we performed a within‐twin‐pair analysis in TwinGene to determine whether the FI‐biomarker associations could be explained by familial confounding (i.e., shared genetic and/or shared environmental factors). Compared to the population‐level estimate of the GlycA‐FI association, the within‐pair estimate was slightly attenuated in dizygotic (DZ) twins and was attenuated to an even greater extent—although not completely—in monozygotic (MZ) twins, indicating potential genetic confounding (Figure 6b). For the creatinine‐FI association, we observed similar effect sizes for the population‐level and within‐pair estimates; however, the wide confidence intervals and statistically nonsignificant estimates in twin pairs precluded us from drawing conclusions on the extent to which this association could be attributable to familial factors (Figure 6d).

## 3 DISCUSSION

We conducted comprehensive observational analyses using data from three population‐based studies, supplemented with MR analyses to explore the effects of circulating metabolic biomarkers on frailty. In the large UKB sample, we found that the vast majority of the 200 assessed biomarkers were significantly associated with frailty, and the directions of associations were mostly consistent for the FI and FP models. Using multivariable linear regression and LASSO models, we selected 59 biomarkers that had the strongest observational associations with the FI in the UKB and replicated 34 of these associations in TwinGene and Health 2000. Specifically, we showed that GlycA was strongly associated with increased FI in both observational and IVW‐MR analyses and across different subgroups. However, the association was at least partly influenced by pleiotropy as indicated by MR‐Egger and co‐twin control analyses. MR analyses also suggested potential causal effects of creatinine, monounsaturated fatty acids, omega‐6, and several cholesterol and lipoprotein traits on the FI, although these effects appeared to be driven by other traits and diseases associated with the exposures. We did not find evidence of causal relationships between the metabolic biomarkers and the FP score.

To the best of our knowledge, this is the first study that has systematically assessed the associations of NMR metabolomic biomarkers with frailty. We reported several novel observational findings, including the strong positive association of GlycA and the generally negative associations of lipoprotein subclasses and components with both the FI and FP scores. Our results for clinical biomarkers are also largely consistent with the literature, which has suggested positive associations of CRP (Velissaris et al., 2017) and glucose (Zaslavsky et al., 2016), and negative associations of vitamin D (Buchebner et al., 2019) and LDL‐cholesterol (Jayanama et al., 2018) with frailty. Since frailty is characterized by the dysregulation in multiple physiological systems such as endocrine system, immune system, brain, and skeletal muscles (Clegg et al., 2013), it is conceivable that frailty is related to many of the circulating metabolomic and clinical biomarkers, which are typically reflective of the current physiological state (Picca et al., 2019).

As observational results can be biased by confounding and reverse causation, we performed two‐sample MR analyses to provide further mechanistic insights into the causal effects of the biomarkers on frailty (Davies et al., 2018). Although the exact biological mechanisms of frailty are not yet fully understood, studies have suggested “inflammaging” (i.e., systemic and chronic inflammation associated with aging) as the converging point of the mechanistic pillars of aging and the main contributor to age‐related diseases, including frailty (Ferrucci & Fabbri, 2018; Franceschi et al., 2018; Kane & Sinclair, 2019; Picca et al., 2022). In line with previous studies showing robust relationships of pro‐inflammatory cytokines such as interleukin‐6 and tumor necrosis factor‐α with frailty (Kane & Sinclair, 2019; Picca et al., 2022), we found that genetically predicted GlycA level was associated with an increased FI. Importantly, this effect was not driven by the individual deficit items included in the FI nor by other traits (CRP and LDL‐cholesterol) associated with GlycA. Meanwhile, our MR results did not support a causal relationship between CRP, a commonly used inflammatory marker in clinical practice (Bassuk et al., 2004), and frailty. GlycA is a novel, NMR‐derived composite biomarker which reflects the concentration and glycosylation of acute‐phase proteins such as α1‐acid glycoprotein, haptoglobin, and α1‐antitrypsin during inflammatory states, and has been proposed as a more sensitive measure than CRP for detecting low‐grade inflammation in younger adults (Chiesa et al., 2022; Connelly et al., 2017). Similarly, a recent study suggested that the interleukin‐6 signaling pathway that also includes CRP, but not CRP itself, could have a causal effect on frailty (Mourtzi et al., 2023). Notably, the MR‐Egger intercept and within‐twin‐pair estimates implicated possible pleiotropic effects. This could be due to the GlycA signal that overlaps with lipoproteins and triglycerides (Connelly et al., 2017), which may also be associated with frailty (Ramsay et al., 2015). Taken together, these findings suggest that GlycA may capture part of the inflammatory response that is causally related to frailty and could be a potential biomarker for early identification and monitoring of frailty. More studies are also warranted to assess the effect of reducing inflammation on frailty due to the currently limited and inconclusive evidence (Espinoza et al., 2022; Orkaby et al., 2021). On the contrary, we observed a putative causal effect of NMR‐derived creatinine on the FI, although this effect was not found in the clinical biomarker serum creatinine, measured using an enzymatic assay. Creatinine is a by‐product of muscle metabolism and increased levels are often indicative of a decline in kidney function (Thongprayoon et al., 2016). As frailty is closely linked to kidney function (Nixon et al., 2018) and has shown to be associated with glomerular filtration rate estimated by serum creatinine (Ballew et al., 2017), the association between creatinine and FI could possibly be explained by kidney disease. This finding was also confirmed in our subgroup analysis, in which we found no statistically significant association between NMR‐derived creatinine and FI in individuals without a chronic kidney disease.

In the observational analyses, we found that most of the lipids and lipoprotein subclasses, except for triglycerides within lipoproteins, were negatively associated with the FI. On the contrary, our MR results indicated that many of these lipid traits, such as subclasses of very low‐, intermediate‐, and low‐density lipoproteins, as well as monounsaturated fatty acids and omega‐6, were associated with an increased FI. These findings are similar to previously reported inverse relationships of total cholesterol and LDL‐cholesterol with frailty in observational studies (Jayanama et al., 2018; Matsuoka et al., 2020), but a positive association between LDL‐cholesterol and the FI in an MR study (Wang et al., 2019). The apparent discrepancy in the direction of the associations could be due to uncontrolled confounders in the observational associations, as well as the different interpretations of the models; the MR estimates represent a lifelong effect of genetically predicted biomarker levels on frailty, while observational estimates usually represent an association over a shorter period of life. Although these biomarkers have shown to be risk factors for cardiovascular diseases and diabetes (Do et al., 2013; Holmes et al., 2018; Richardson et al., 2020; White et al., 2016; Zagkos et al., 2022), some studies have also found no or inverse relationship between LDL‐cholesterol and mortality risk among older adults (Butterworth et al., 2009; Li et al., 2021). Moreover, MR estimates could potentially be biased by SNPs that have pleiotropic effects. It has been shown that SNPs in lipid‐associated genes, such as *PCSK9*, are highly pleiotropic and are robustly associated with various lipoprotein subclasses, cholesterols, as well as omega‐6 fatty acids and sphingomyelin (Würtz et al., 2013). Importantly, when the FI was stripped of cardiometabolic items such as heart failure, stroke, and diabetes, the MR estimates for all these lipid and lipoprotein traits attenuated to null, suggesting that their effects on the FI are likely mediated by cardiometabolic diseases. This also highlights the importance of optimizing cardiovascular disease risk factors in mitigating frailty (Atkins et al., 2019).

We used both the FI and FP to measure frailty, which are the two most widely validated frailty measures in community‐dwelling older adults (Dent et al., 2016). Despite being different operational approaches to frailty, previous research has demonstrated that the FI and FP share a large part of their genetic and environmental etiologies, and may thus tap the same root causes of frailty (Livshits et al., 2018). Although most biomarkers had similar effects on the FI and FP in the observational analysis, we did not observe any statistically significant association between biomarkers and the FP in the main MR analysis. This could be due to differences in their underlying mechanisms, in which the FI is a multidimensional construct that incorporates deficits from multiple tissue and organ systems (Searle et al., 2008), while the FP is more related to physical functioning and defines a clinical syndrome that emerges from a decline in physiological reserves (Fried et al., 2001). Therefore, biomarkers directly related to the FI items, such as lipids and lipoproteins that are indicative of cardiometabolic health, may be more strongly associated with the FI than the FP. Of note, in the sensitivity MR analysis, we found that GlycA was significantly associated with both the FI and FP after excluding the potentially pleiotropic SNPs, indicating that inflammation could be a common driver of both the multidimensional (FI) and physical (FP) frailty. Moreover, while the FP was originally developed for older adults, the continuous FI is often more informative on the frailty status in younger adults (Clegg et al., 2013). The null association for the FP could be explained by the relatively young and healthy population of UKB and the low prevalence of frailty as measured by the FP, which may have reduced statistical power in the GWAS and the subsequent MR analysis. The FP was also not available in TwinGene and Health 2000. More studies are therefore needed to confirm the relationships between metabolic biomarkers and physical frailty.

The strengths of this study include the use of a standardized NMR metabolomics platform in three large cohorts to identify metabolic biomarkers that have strong evidence of associations with the two measures of frailty. This platform allowed us to examine novel biomarkers, such as inflammatory markers and lipids that are not yet commonly assessed in clinical practice. Using the MR and co‐twin control methods, we were also able to make causal inferences. However, some limitations should be considered when interpreting our results. Firstly, our analysis was restricted to samples with European ancestry. Although it minimized bias arising from population stratification, it could also reduce generalizability to other ethnic groups. Secondly, our metabolomics and GWAS data mainly relied on UKB, which provided us with enough statistical power but may not be representative to the general population due to the healthy selection (Fry et al., 2017). Thirdly, because of the cross‐sectional design of the included observational studies, we were unable to delineate longitudinal relationships between biomarkers and changes in frailty levels. Fourthly, our observational and two‐sample MR analyses only assumed linear relationships. Whether there may be nonlinear associations between metabolic biomarkers and frailty need to be examined in future studies. Finally, although the NMR metabolomics platform from Nightingale Health provides a comprehensive and standardized assessment of circulating metabolites, it does not capture the whole blood metabolome and includes only a limited number of metabolites. Some of the NMR biomarkers also lack specificity and are associated with a wide range of diseases (Julkunen et al., 2023). Hence, further investigation is needed to examine if other metabolites may also be related to frailty.

In conclusion, our results show that a large proportion of the blood metabolome is associated with frailty. We also present evidence of the potential causal effects of GlycA, creatinine, and several lipid traits on the FI. These findings provide novel insights into the metabolic underpinnings of frailty and outline the foundations for the continuing search of specific biomarkers that can facilitate early identification and management of frailty.

## 4 METHODS

### 4.1 Study population

The UKB was used as the discovery cohort. It is a population‐based, cross‐sectional study with half a million adults aged 37–73 years recruited across the UK between 2006 and 2010 (Sudlow et al., 2015). At baseline assessment, participants provided biological samples and other health‐related data via touch screen questionnaires and physical measurements in one of the 22 assessment centers across England, Wales, and Scotland. For the observational analysis, we excluded UKB individuals who had withdrawn from the study (*n* = 172), had missing data on the FI (*n* = 2293) and were self‐reported as non‐white ethnicity (*n* = 28,260). From the 471,906 eligible UKB participants, we then selected two subsamples for the analyses of the NMR metabolomic biomarkers and clinical biomarkers, respectively. In the first subsample of 104,378 participants who had complete data on the 168 metabolomic biomarkers, we further removed samples that failed quality control (i.e., labeled as “high lactate,” “high pyruvate,” “low glucose,” or “low protein”), yielding *n* = 90,573 individuals in the analysis. The second subsample consisted of 67,488 participants who had complete data on the 32 clinical biomarkers.

Replication was conducted in two independent studies: the Swedish TwinGene study and the Finnish Health 2000 Survey. TwinGene is a subcohort study within the Swedish Twin Registry, which collected blood sample from 12,648 older Swedish twins in 2004–2008 (Magnusson et al., 2013). Participants had previously participated in a telephone interview survey, the Screening Across the Lifespan Twin (SALT) study in 1998–2002. The Finnish Health 2000 Survey is a nationally representative survey conducted during 2000–2001, and its two‐stage stratified cluster sample consisted of 8028 persons aged ≥30 (Heistaro, 2008). The Health 2000 Survey included self‐administered questionnaires, interviews, health examinations, and laboratory measurements. The participation rate in the health examination was 85%. Using the same data processing procedures as in the UKB, we excluded TwinGene and Health 2000 participants who had missing data on the FI, or any of the metabolomic or clinical biomarkers, and failed quality control, yielding a total of 11,025 TwinGene participants and 6073 Health 2000 participants in the analysis.

The UKB study had an ethical approval from the North West Multi‐Centre Research Ethics Committee. The TwinGene study was approved by the Regional Ethics Review Board in Stockholm. The Health 2000 Survey was approved by the Ethical Committee for Research in Epidemiology and Public Health at the Hospital District of Helsinki and Uusimaa. A written informed consent was obtained from all participants.

### 4.2 Metabolic biomarker profiling

Circulating metabolomic biomarkers were measured using a targeted high‐throughput NMR metabolomics platform (Nightingale Health Ltd., Helsinki, Finland). Details on the first data release of the NMR metabolomic biomarkers in UKB have been described elsewhere (Julkunen et al., 2023). Briefly, 168 metabolic measures were quantified from a random subset of 118,461 nonfasting baseline EDTA plasma samples. These biomarkers include clinically validated biomarkers, such as cholesterols, fatty acids, amino acids, and inflammation markers, as well as other emerging biomarkers such as lipoprotein subclasses. The 81 ratios derived from combinations of the 168 measured biomarkers were not included in this analysis due to unclear biological interpretations. The same 168 biomarkers were quantified from serum samples in the TwinGene and Health 2000 participants, who had been instructed to fast overnight before blood collection.

Additionally, we studied 32 clinical biomarkers from serum and urine samples of UKB participants (rheumatoid factor and estradiol were not included due to high missingness). These biomarkers are diagnostic measures or risk factors for diseases, including cardiovascular, bone and joint, cancer, diabetes, renal, and liver‐related biomarkers. Details of the assay methods and quality control are described on the UKB website (https://biobank.ndph.ox.ac.uk/showcase/showcase/docs/serum_biochemistry.pdf and http://biobank.ctsu.ox.ac.uk/crystal/crystal/docs/urine_assay.pdf). Details of the clinical biomarkers in TwinGene and Health 2000 have been described previously (Kananen et al., 2023; Li et al., 2021).

Descriptive statistics of all the included biomarkers are summarized in Table S1. Spearman's correlations between the metabolic biomarkers were visualized as heatmaps. To enable comparison of effect sizes for biomarkers with different units and concentration ranges, we standardized all biomarkers to have mean = 0 and SD = 1 within each sample.

### 4.3 Frailty measures

An FI has previously been created and validated in the UKB using 49 self‐reported deficit items from 11 categories (Williams et al., 2019), covering a range of diseases, signs, and symptoms from physiological and mental domains (items are shown in Table S4). After excluding individuals who had >20% missing data across the 49 items, we calculated the FI as the sum of deficit divided by the total number of nonmissing items in each individual. The FIs in TwinGene (44‐item, collected at baseline of the SALT study) (Li et al., 2019) and Health 2000 (38‐item) were constructed using the same procedure. In all analyses, we multiplied the FI by 100 and considered it as the percentage of deficit accumulation (0%–100%).

The FP was used as a secondary outcome in a sensitivity analysis in the UKB. Based on the five frailty criteria in the Fried model (Fried et al., 2001), a modified FP has previously been constructed in the UKB (Hanlon et al., 2018). Weight loss, exhaustion, slowness, and low activity were assessed by self‐reported questionnaire items, whereas weakness was assessed by the measured grip strength at baseline (scoring of the items are described in Table S5). An FP score was then calculated as the number of frailty criteria present in an individual. Although the FP is commonly categorized into three groups for assessing frailty in older adults aged ≥65 years (Fried et al., 2001), we considered it as ordinal variable from 0 to 5 to maximize statistical power, due to the relatively young age (mean 56.8 years) and low prevalence of frailty in UKB (only ~3% of UKB participants were deemed frail by the FP).

### 4.4 Statistical analysis

#### 4.4.1 Identification of frailty‐associated biomarkers in UKB

In the discovery phase in UKB, we applied two approaches to identify frailty‐associated metabolic biomarkers:

1. Multivariable linear regression of FI on each biomarker. The base models were adjusted for age (continuous) and sex, and the fully adjusted models were additionally adjusted for baseline assessment centers (England, Wales, and Scotland), BMI (continuous), smoking (never, previous, and current), alcohol consumption (less than 3 times a month, 1 to 4 times a week, daily or almost daily), education level (low, intermediate, and high), and Townsend deprivation index (continuous). All the estimates were calculated as *β*‐coefficients per SD increase in biomarker values. To account for multiple testing of up to 200 biomarkers in the large sample of UKB, we applied the stringent Bonferroni correction and considered *p* < 0.00025 (i.e., 0.05/200) as statistically significant.
2. Feature selection using LASSO. Due to the large number of metabolites and their high degree of collinearity, we applied the LASSO procedure (Tibshirani, 2011) to select the independent and most dominant biomarkers that contributed to the variance of the FI, as well as to minimize overfitting. Using FI as the dependent variable, we fitted two LASSO linear regression models, one including 168 metabolomic biomarkers, and the other 32 clinical biomarkers as the explanatory variables. Age and sex were also included in both models. We used a 10‐fold cross validation to optimize the λ regularization parameter. Estimates of the noninformative features were then shrunk to zero, based on the λ value that gave a mean squared error within 1 standard error of the minimum (Figure S6).

As a sensitivity analysis, we used the FP as the outcome in linear regression models and compared the direction of the biomarker‐FI and biomarker‐FP associations. To examine whether the associations were influenced by outlier biomarker values, we repeated the linear regression analysis after excluding values outside 5 interquartile ranges from the median. Furthermore, we performed subgroup analyses of the biomarker‐FI associations by age at baseline (<60 vs. ≥60 years) and sex (women vs. men). As our main analyses were constrained to white UKB participants, we also repeated the analysis in non‐white ethnic groups to test if the associations differ by ethnicity. Finally, as MR implicated potential causal effects of GlycA and creatinine on the FI, we additionally stratified the observational analyses between GlycA and FI by CRP and LDL‐cholesterol categories, and between creatinine and FI by chronic kidney disease to examine whether the associations may be influenced by their related traits (Connelly et al., 2017; Levey et al., 2009).

#### 4.4.2 Replication in TwinGene and health 2000

For the biomarkers that (i) passed the Bonferroni‐corrected threshold (*p* < 0.00025) in multivariable linear regression models and (ii) had nonzero coefficients from LASSO models, we performed replication in TwinGene and Health 2000 using FI as the outcome. Associations were assessed using linear regression models adjusted for age, sex, BMI, smoking, education, and alcohol consumption. The models in TwinGene were also accounted for twin relatedness (i.e., cluster‐robust standard errors). Results from the replication cohorts were meta‐analyzed using a DerSimonian‐Laird random‐effects model (DerSimonian & Laird, 1986), where associations with *p* < 0.05 were considered as “replicated”. The biomarkers that were replicated or unavailable in replication cohort were then proceeded to MR analyses.

#### 4.4.3 Mendelian randomization

Two‐sample MR analyses were performed to investigate causal relationships between the selected metabolites and frailty. Only genetic data on European‐ancestry individuals were included to ensure comparability of the SNPs. Details on the datasets used are provided in Table S11. Briefly, SNPs associated with the biomarkers (exposures) were taken from the largest available GWASs, namely UKB (*n* = 115,078, for NMR metabolomic biomarkers) (Borges et al., 2022), Meta‐Analyses of Glucose and Insulin‐related traits Consortium (*n* = 123,665, for HbA1c) (Wheeler et al., 2017), Cohorts for Heart and Aging Research in Genomic Epidemiology Consortium (*n* = 204,402, for CRP) (Ligthart et al., 2018), and Global Lipids Genetics Consortium (*n* = 187,365, for total cholesterol, LDL‐cholesterol, and triglycerides) (Willer et al., 2013). For the remaining clinical biomarkers, we performed a GWAS in a randomly selected 50% of the UKB sample who were eligible and passed quality control (*n* = up to 204,402; excluded individuals with non‐European ancestry, consent withdrawal, sex chromosome aneuploidy, extreme heterozygosity or missingness, and without genotype and phenotype data). A mixed linear model‐based GWAS analysis (“fastGWA”) was used, which is an efficient method to control for relatedness between individuals by a sparse genetic relationship matrix (Jiang et al., 2019). Age, sex, genotyping array, and the first 10 principal components were included as covariates. Following the same pipeline, we performed a GWAS for the FI and FP in UKB to obtain summary statistics for the SNP‐frailty (outcome) associations. The GWAS for frailty was performed in UKB subsamples that did not overlap with the exposure GWASs to avoid biasing the two‐sample MR analysis (Burgess et al., 2016).

To obtain valid causal estimates in MR, genetic variants that are used as IVs should fulfill three assumptions: (i) they are robustly associated with the exposure (relevance), (ii) they are independent of any confounders (independence), and (iii) they affect the outcome only through the exposure (exclusion restriction) (Davies et al., 2018). We selected SNPs as IVs if they were associated with the biomarker of interest at a genome‐wide significance level (*p* < 5 × 10^−8^) and were not in linkage disequilibrium with other SNPs (*r* ^2^ < 0.001 within a clumping window of 10,000 kb). Palindromic SNPs with minor allele frequency >0.42 or SNPs not available in the outcome GWASs were excluded. Instrument strength was evaluated by the *F*‐statistic (Davies et al., 2018). The multiplicative random‐effects IVW‐MR method was applied as the primary approach, which provides unbiased estimates if all the IVs are valid or if the overall pleiotropy is balanced to be zero (Burgess et al., 2013). The Cochran's *Q* test was used to assess heterogeneity across the IVs. To correct the main IVW results for multiple testing (a total of 49 biomarkers × 2 frailty measures = 98 tests), we applied a 5% FDR correction (Benjamini & Hochberg, 1995) and considered *p* < 0.011 as statistically significant. To test for robustness of our results, we conducted MR analyses that relax assumptions on horizontal pleiotropy: (i) MR‐Egger, which allows for pleiotropic effects under the instrument strength independent of direct effect (InSIDE) assumption, with an intercept term indicating the average pleiotropic effect (Bowden et al., 2015); (ii) weighted median, which assumes over 50% of the IVs are valid (Bowden et al., 2016); (iii) weighted mode, which assumes a plurality of IVs are valid (Hartwig et al., 2017); and (iv) MR‐PRESSO, which detects and corrects for horizontal pleiotropy by excluding outliers (Verbanck et al., 2018). As a sensitivity analysis, we used a smaller set of SNPs that were not associated with other metabolomic biomarkers at genome‐wide significance as the IVs (i.e., excluding potentially pleiotropic SNPs). Finally, to examine if the observed associations were driven by the individual FI items, we repeated the MR analysis using 11 modified FIs that were stripped of items from each category as the outcomes (Table S4).

#### 4.4.4 Co‐twin control analysis in TwinGene

Taking the advantage of twin data in TwinGene, we employed the co‐twin control method to elucidate whether a biomarker‐FI association may be attributable to familial confounding (shared genetic or shared environmental factors) (McGue et al., 2010). This method assumes that MZ and DZ twins share 100% and ~ 50% of their segregating genes, respectively, and that both MZ and DZ twins share the same family environment. If the association is not influenced by familial influences (i.e., in line with a causal hypothesis), the effect sizes should remain similar across the population‐level and within‐pair estimates. If the association is explained by shared genetic factors (pleiotropy), we would expect an attenuation of the estimate to null within MZ twin pairs, while the estimate within DZ twin pairs is expected to lie between the population‐level and MZ estimates. If the association is explained by shared environmental factors, a similar attenuation would be expected in both DZ and MZ twins. Conditional generalized estimating equation models were used to obtain within‐twin‐pair estimates. All models were adjusted for age, sex, BMI, smoking, alcohol consumption, and years of education.

## AUTHOR CONTRIBUTIONS

Jonathan K. L. Mak and Juulia Jylhävä contributed to the study concept and design. Jonathan K. L. Mak, Laura Kananen, Yunzhang Wang, Tuija Jääskeläinen, Seppo Koskinen, and Patrik K. E. Magnusson contributed to data curation. Jonathan K. L. Mak, Laura Kananen, Chenxi Qin, Ralf Kuja‐Halkola, Bowen Tang, Jake Lin, Yi Lu, Sara Hägg, and Juulia Jylhävä were involved in methodology. Jonathan K. L. Mak, Laura Kananen, and Chenxi Qin performed statistical analysis. Sara Hägg, Juulia Jylhävä, and Laura Kananen were involved in funding acquisition. Ralf Kuja‐Halkola, Sara Hägg, and Juulia Jylhävä were involved in supervision. Jonathan K. L. Mak and Juulia Jylhävä drafted the manuscript. All authors participated in writing and reviewing of the manuscript.

## FUNDING INFORMATION

This work was supported by the Swedish Research Council (2018–02077, 2019–01272, 2020–06101, 2022–01608), the Academy of Finland (grant no. 349335), the Sigrid Jusélius Foundation, the Loo & Hans Osterman Foundation, the Strategic Research Program in Epidemiology at Karolinska Institutet, the Karolinska Institutet Foundations, the King Gustaf V and Queen Victoria's Foundation of Freemasons, the Yrjö Jahnsson Foundation (grant no 20217416), the Juho Vainio Foundation (grant number 202100335), and the Päivikki and Sakari Sohlberg Foundation (grant number 220032).

## CONFLICT OF INTEREST STATEMENT

The authors declare no conflict of interest.

## CODE AVAILABILITY

All the data processing, visualization, and statistical analyses were performed using R v.4.1.3 (R Foundation for Statistical Computing, Vienna, Austria; https://www.r‐project.org/). Heatmaps were created using the R package *ggcorrplot* (v.0.1.3; https://cran.r‐project.org/web/packages/ggcorrplot/). Circos plots were created using the R packages *circlize* (v.0.4.15; https://github.com/jokergoo/circlize) and *EpiViz* (v.0.0.0.9000; https://github.com/mattlee821/EpiViz). Forest plots were created using the R package *ggforestplot* (v.0.1.0; https://nightingalehealth.github.io/ggforestplot/). LASSO models were fitted using the R package *glmnet* (v.4.1–4; https://glmnet.stanford.edu/). Generalized estimating equation models were fitted using the R package *drgee* (v.1.1.10; https://cran.r‐project.org/web/packages/drgee/). Meta‐analysis was performed using the R package *metfor* (v.3.8–1; https://www.metafor‐project.org/). MR analyses were performed using the R packages *TwoSampleMR* (v.0.5.6; https://mrcieu.github.io/TwoSampleMR/) and *MR‐PRESSO* (v.1.0; https://github.com/rondolab/MR‐PRESSO). All codes used in data preparation and analysis are publicly available at https://github.com/jonathanklmak/Metabolomics_frailty/.

## Supporting information


Figures S1–S7

Tables S1–S18

## Data Availability

Individual‐level data cannot be stored in public repositories or otherwise made publicly available due to ethical and data protection restrictions. However, data are available upon request for researchers who meet the criteria for access to confidential data. Data from the UK Biobank are available to bona fide researchers upon application at https://www.ukbiobank.ac.uk/enable‐your‐research. Data from the TwinGene study are available through an application procedure at the Swedish Twin Registry. (http://ki.se/en/research/the‐swedish‐twin‐registry). The Health 2000 data are available from the Finnish Institute for Health and Welfare (THL) based on a research collaboration agreement and permission to handle the confidential data (contact: terveys-2000-2011@thl.fi). All the supporting data for the MR analyses are available within this article and the Supplementary Information. GWAS summary statistics for the NMR metabolomics biomarkers, CRP, total cholesterol, HbA1c, LDL‐C, and triglycerides used in the MR analyses are publicly available at https://gwas.mrcieu.ac.uk/ and https://magicinvestigators.org/downloads/, as detailed in Table S11. Further data are available upon reasonable request to the corresponding author.
