# Supplementary material for: Unraveling the metabolic underpinnings of frailty using multicohort observational and Mendelian randomization analyses
Source: Aging Cell. 2023 May 15;22(8):e13868. doi: 10.1111/acel.13868 (PMC10410014; doi:10.1111/acel.13868)
Supplement: Supplementary file 1 — Figures S1–S7 [file ACEL-22-e13868-s002.pdf]

Unraveling the metabolic underpinnings of frailty using multi-cohort observational and Mendelian randomization analyses

Jonathan K. L. Mak[1]*, Laura Kananen[1,2], Chenxi Qin[1], Ralf Kuja-Halkola[1], Bowen Tang[1], Jake Lin[1,2,3], Yunzhang Wang[1,4], Tuija Jääskeläinen[5], Seppo Koskinen[5], Yi Lu[1,6], Patrik K. E. Magnusson[1], Sara Hägg[1], Juulia Jylhävä[1,2]

[1] Department of Medical Epidemiology and Biostatistics, Karolinska Institutet, Stockholm, Sweden
[2] Faculty of Social Sciences (Health Sciences) and Gerontology Research Center (GEREC), University of Tampere, Tampere, Finland
[3] Institute for Molecular Medicine Finland FIMM, Helsinki Institute of Life Science HiLIFE, University of Helsinki, Helsinki, Finland
[4] Department of Clinical Sciences, Danderyd Hospital, Karolinska Institutet, Stockholm, Sweden
[5] Finnish Institute for Health and Welfare, Helsinki, Finland
[6] Department of Global Public Health, Karolinska Institutet, Stockholm, Sweden

* Correspondence: Jonathan K. L. Mak, Department of Medical Epidemiology and Biostatistics, Karolinska Institutet, Nobels väg 12A, 171 77 Stockholm, Sweden. Email: jonathan.mak@ki.se

Supplementary Figures

Supplementary Figure 1. Distribution of the 168 NMR metabolomic biomarkers in the UK Biobank (n=90,573) 2

Supplementary Figure 2. Distribution of the 32 clinical biomarkers in the UK Biobank (n=67,488) .. 4

Supplementary Figure 3. Heatmap of the Spearman correlations between the 168 NMR metabolomic biomarkers in the UK Biobank (n=90,573) 5

Supplementary Figure 4. Heatmap of the Spearman correlations between the 32 clinical biomarkers in the UK Biobank (n=67,488) 6

Supplementary Figure 5. Fully adjusted observational associations of the metabolic biomarkers with FI and FP scores in the UK Biobank 7

Supplementary Figure 6 Cross-validated mean-squared error of the LASSO models for the 168 NMR metabolomic biomarkers (n=90,573) and the 32 clinical biomarkers (n=67,488) in the UK Biobank 8

Supplementary Figure 7. Associations between metabolic biomarkers and FI in the UK Biobank, stratified by age groups, sex, and in non-white ethnic groups 9

Supplementary Figure 1. Distribution of the 168 NMR metabolomic biomarkers in the UK Biobank (n=90,573)

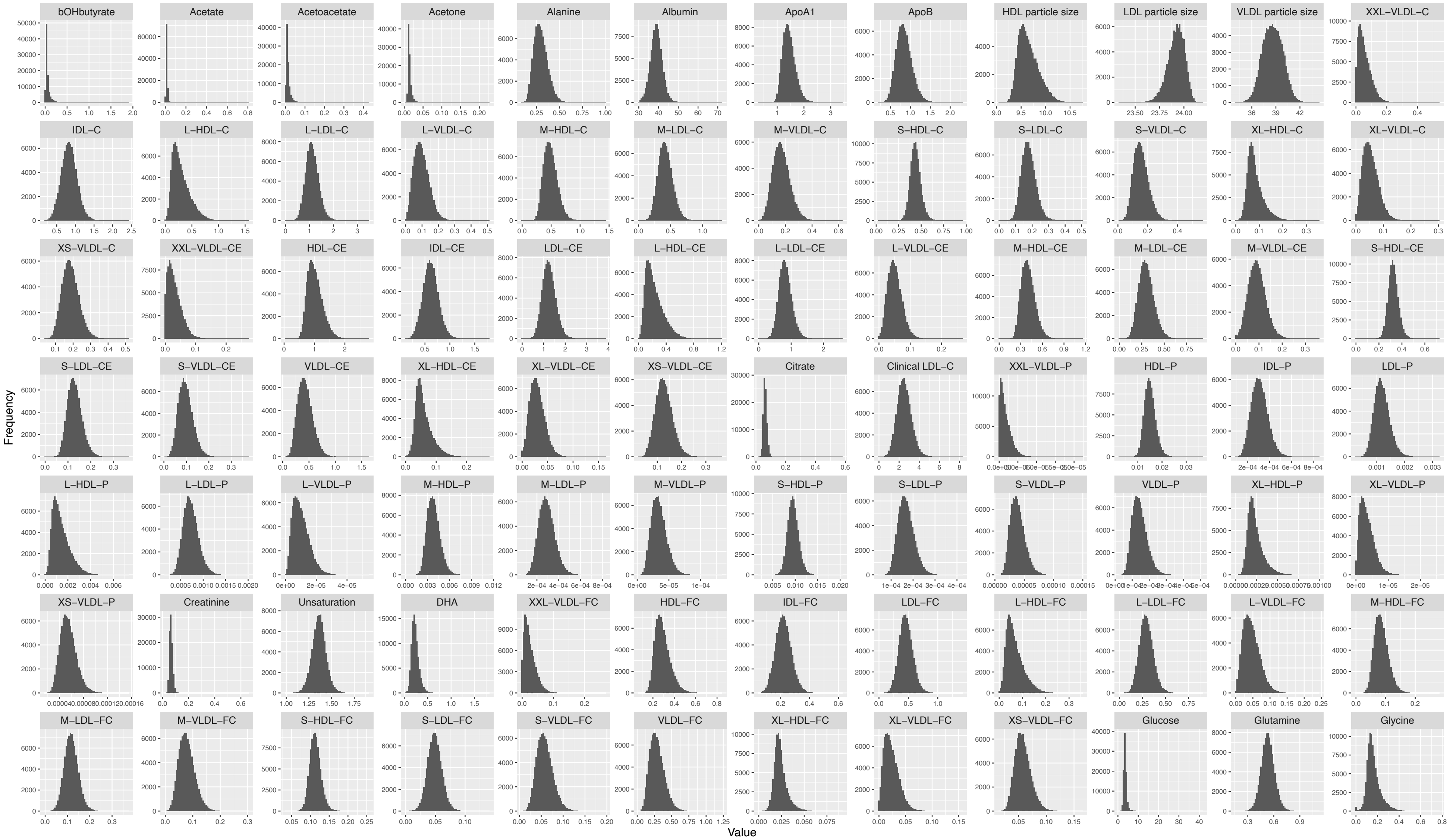

Supplementary Figure 1. *(continued)*

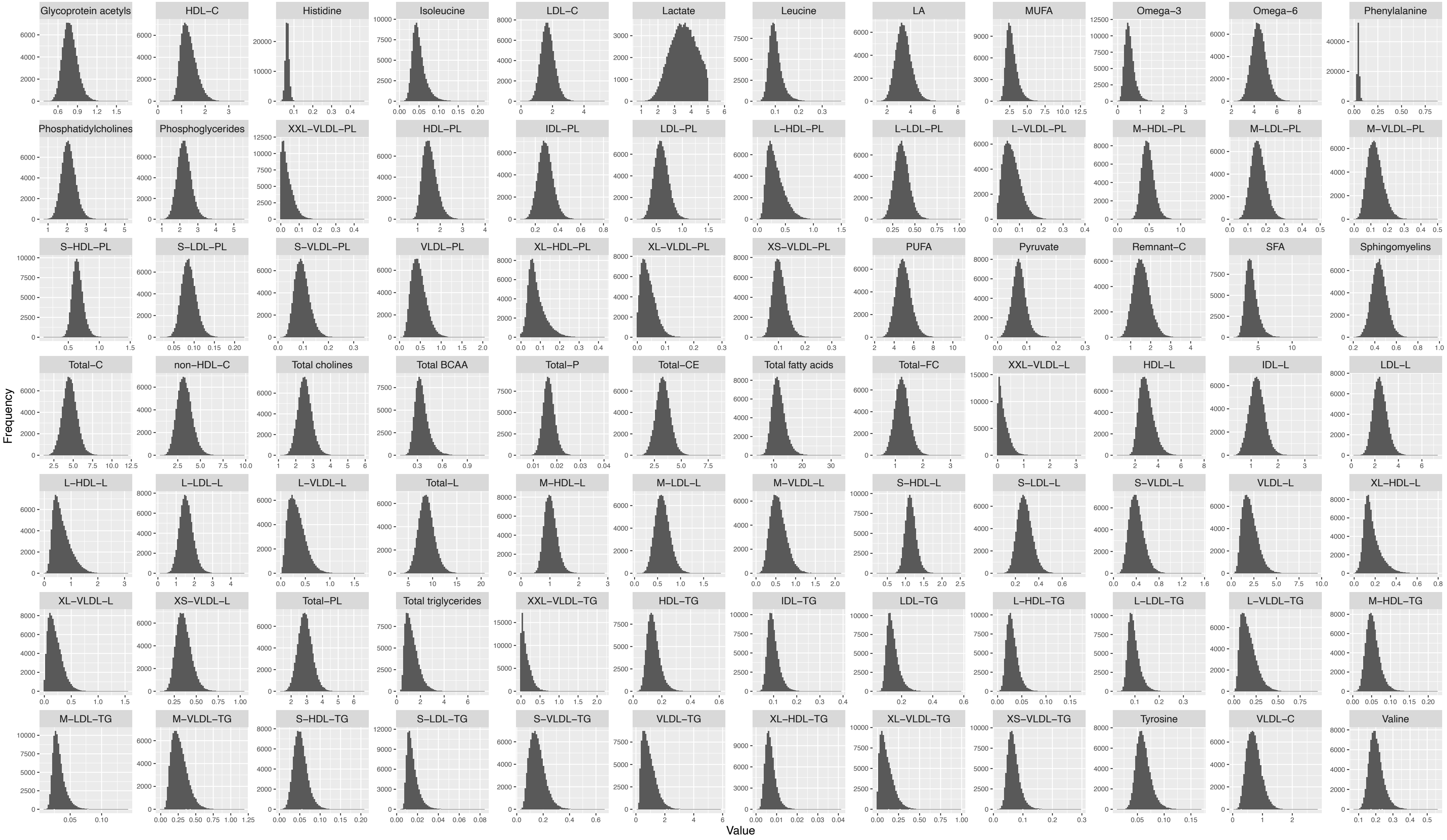

Only individuals with complete data on the 168 NMR metabolomic biomarkers were included. NMR, nuclear magnetic resonance.

Supplementary Figure 2. Distribution of the 32 clinical biomarkers in the UK Biobank (n=67,488)

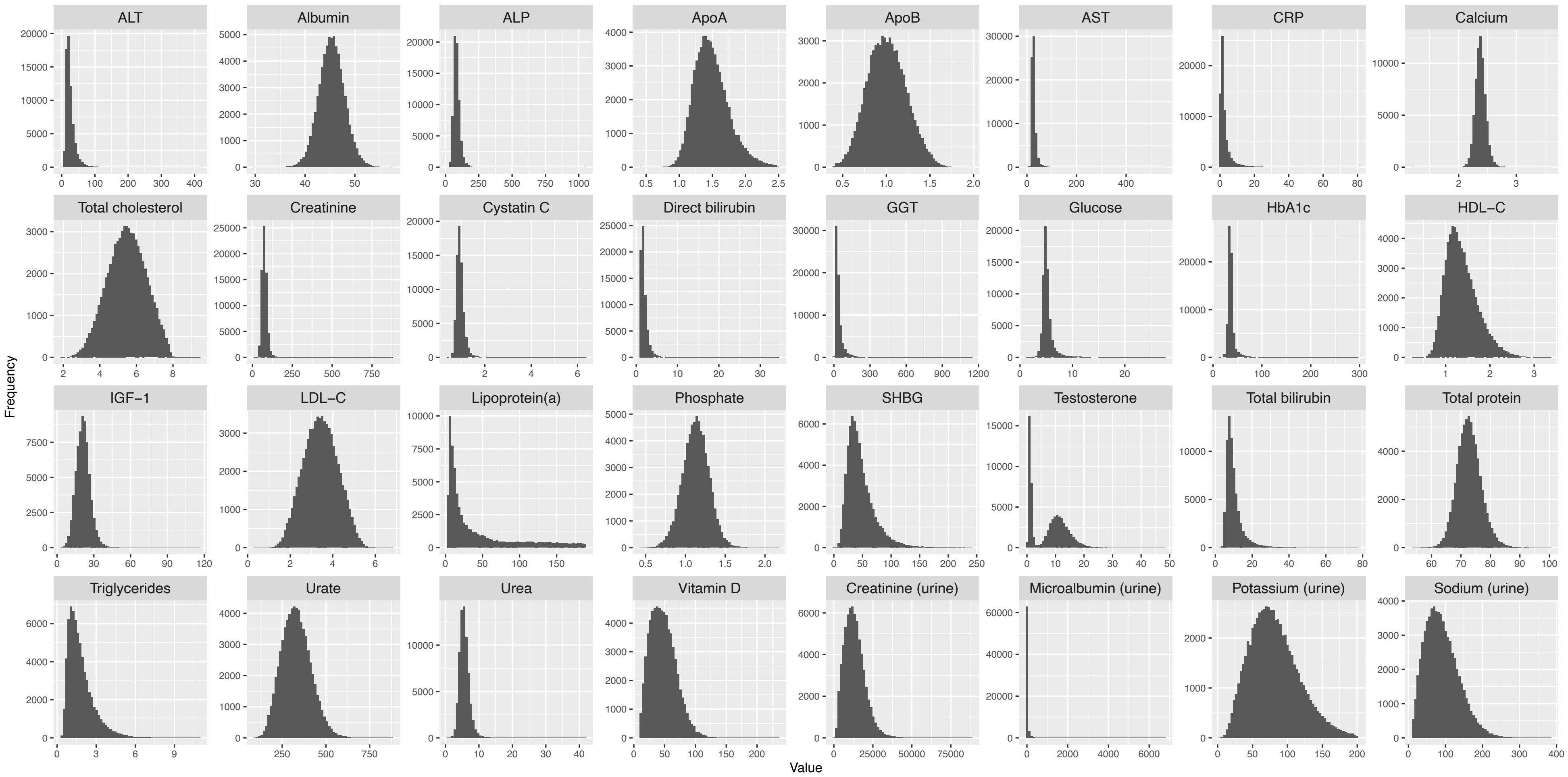

Only individuals with complete data on the 32 clinical biomarkers were included. NMR, nuclear magnetic resonance.

Supplementary Figure 3. Heatmap of the Spearman correlations between the 168 NMR metabolomic biomarkers in the UK Biobank (n=90,573)

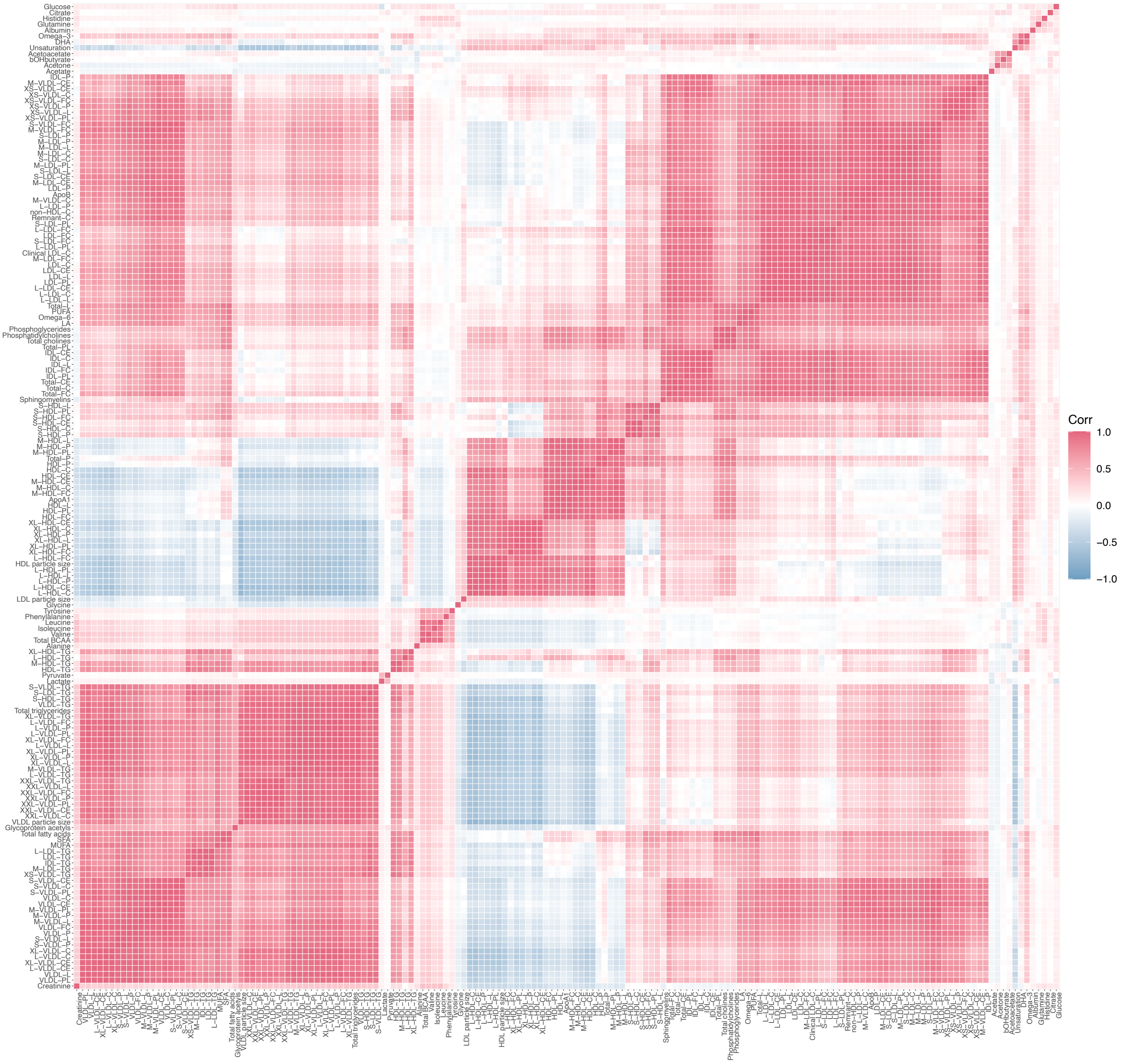

Notes: Only individuals with complete data on the 168 NMR metabolomic biomarkers were included. Each square represents the Spearman's correlation coefficient between the two corresponding biomarkers. Biomarkers are ordered by hierarchical clustering. The corresponding numeric correlation coefficients are presented in **Supplementary Table 2**. NMR, nuclear magnetic resonance.

Supplementary Figure 4. Heatmap of the Spearman correlations between the 32 clinical biomarkers in the UK Biobank (n=67,488)

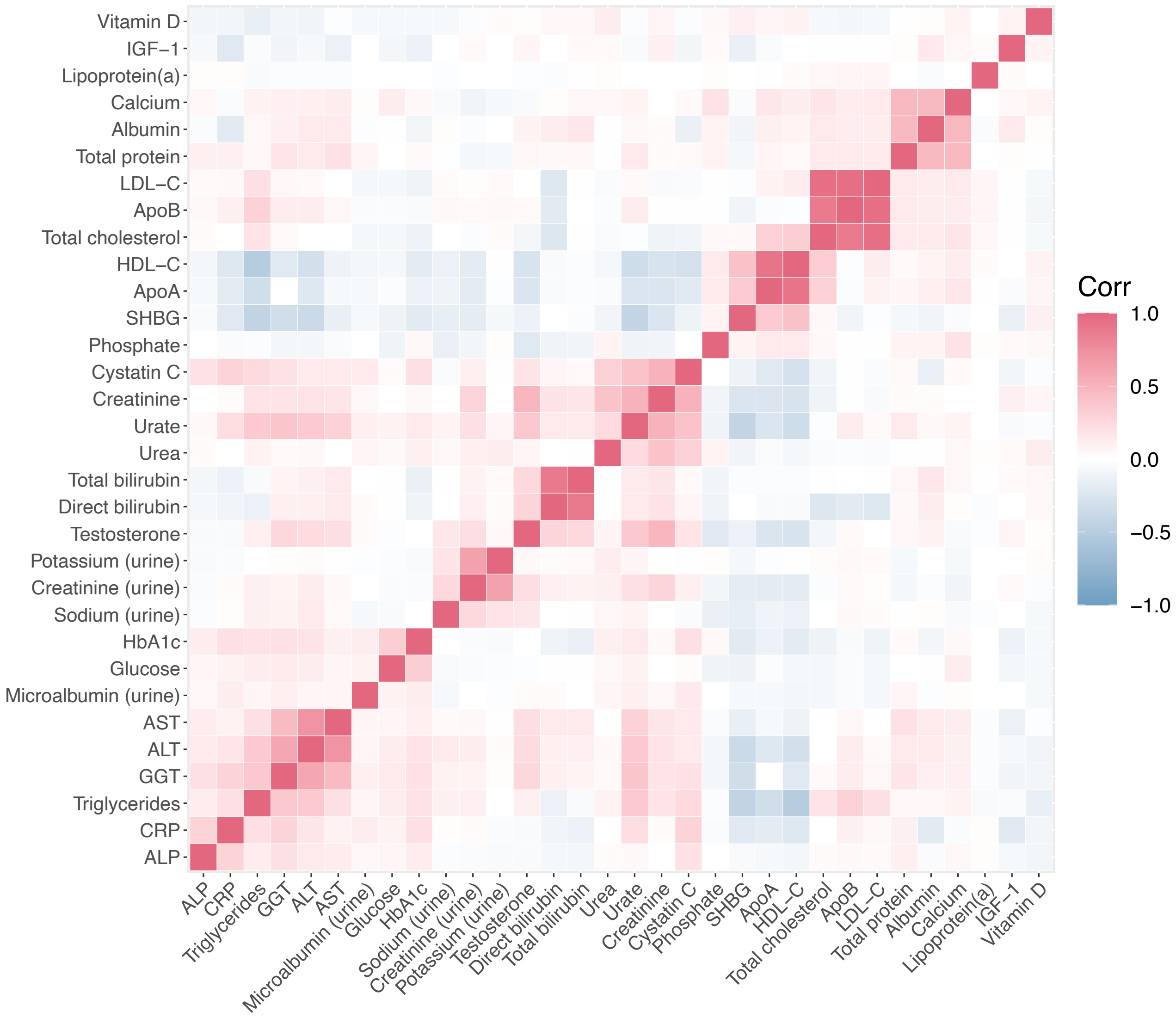

Notes: Only individuals with complete data on the 32 clinical biomarkers were included. Each square represents the Spearman's correlation coefficient between the two corresponding biomarkers. Biomarkers are ordered by hierarchical clustering. The corresponding numeric correlation coefficients are presented in **Supplementary Table 3**.

Supplementary Figure 5. Fully adjusted observational associations of the metabolic biomarkers with FI and FP scores in the UK Biobank

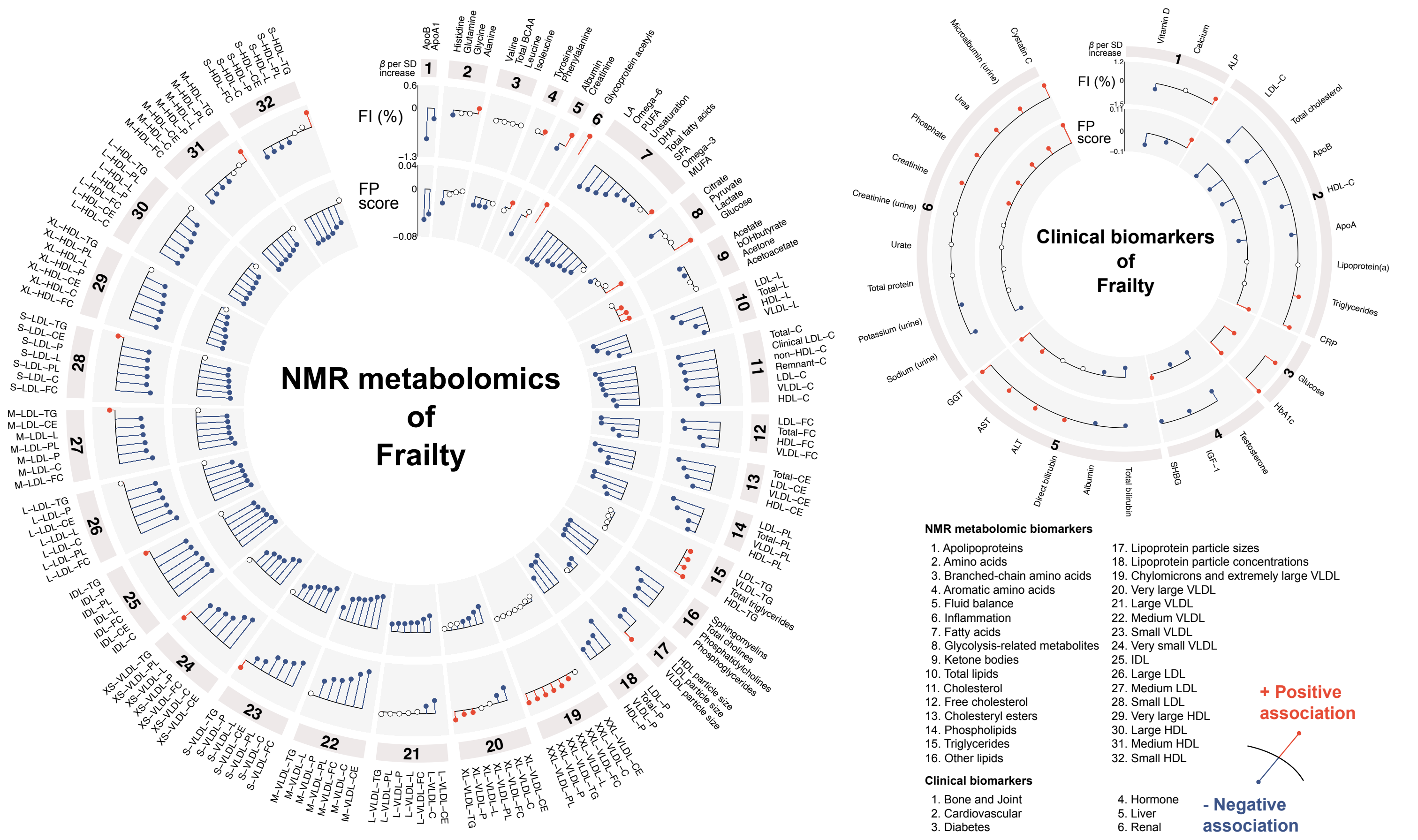

Notes: Estimates represent the changes in frailty index (outer tracks of the circles) or frailty phenotype score (inner tracks of the circles) per 1 standard deviation increase in the biomarker level, adjusted for age, sex, baseline assessment center (England, Wales, Scotland), body mass index (continuous), smoking (never, previous, current), alcohol consumption (less than 3 times a month, 1–4 times a week, daily or almost daily), education level (high, intermediate, low), and deprivation index. Red dots indicate positive associations, whereas blue dots indicate negative associations. Filled dots represent statistically significant associations after Bonferroni correction at p<.00025 (i.e., .05/200, considering 200 biomarkers). The corresponding numeric estimates are shown in **Supplementary Tables 6–7**. ALP, alkaline phosphatase; ALT, alanine aminotransferase; AST, aspartate aminotransferase; ApoA, apolipoprotein A; ApoB, apolipoprotein B; BCAA, branched-chain amino acids; bOHbutyrate, 3-hydroxybutyrate; C, cholesterol; CE, cholesteryl esters; DHA, docosahexaenoic acid; FC, free cholesterol; FI, frailty index; FP, frailty phenotype; GGT, gamma glutamyltransferase; HbA1c, glycated hemoglobin; HDL, high-density lipoproteins; IDL, intermediate-density lipoproteins; IGF, insulin-like growth factor; L, large (when used as prefix) or total lipids (when used as suffix); LA, linoleic acid; LDL, low-density lipoproteins; M, medium; MUFA, monounsaturated fatty acids; NMR, nuclear magnetic resonance; P, particle concentrations; PL, phospholipids; PUFA, polyunsaturated fatty acids; S, small; SD, standard deviation; SFA, saturated fatty acids; SHBG, sex hormone binding globulin; TG, triglycerides; Unsaturation, degree of unsaturation; UKB, UK Biobank; VLDL, very low-density lipoproteins; XL, very large; XS, very small; XXL, extremely large.

Supplementary Figure 6 Cross-validated mean-squared error of the LASSO models for the 168 NMR metabolomic biomarkers (n=90,573) and the 32 clinical biomarkers (n=67,488) in the UK Biobank

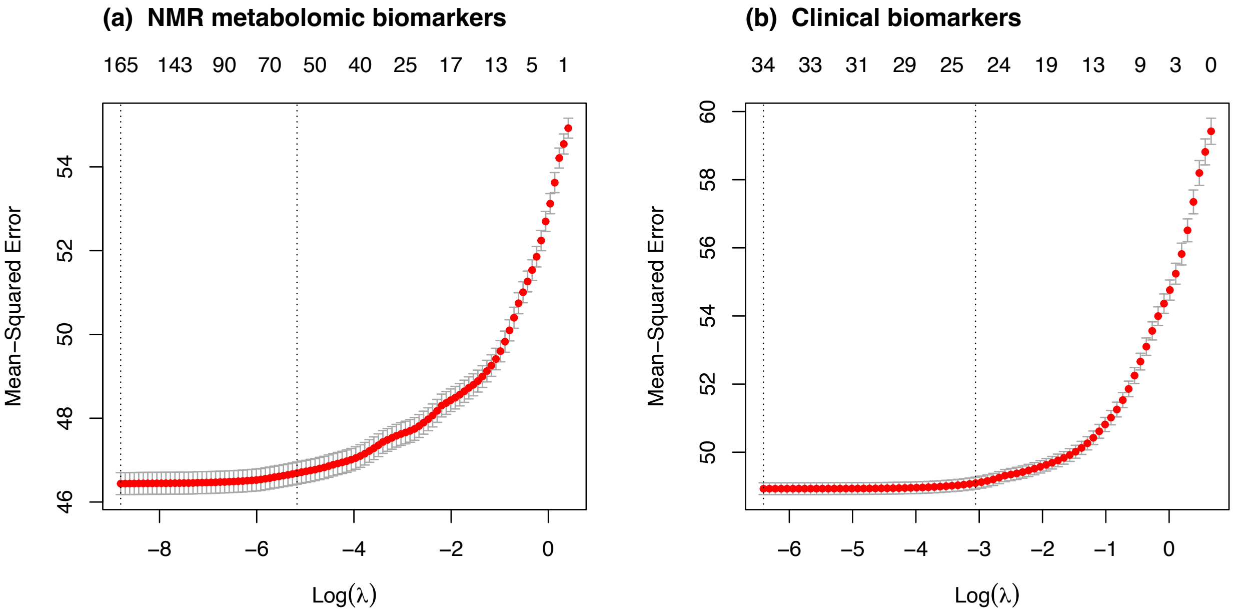

Notes: Age and sex were included in both models, and the frailty index (FI) was used as the dependent variable. In each panel, the red dots represent the mean-squared error (MSE) at each λ value, along with the 95% confidence interval. In each panel, the left dotted vertical line indicates the λ value that minimizes the cross-validated MSE, whereas the right vertical line indicates the highest value of MSE within one standard error of the minimum error. The numbers above the graph represent the number of variables retained at each given λ value. In our analysis, we selected the λ values that gave the MSE within one standard error of the minimum (to increase shrinkage), which were λ=0.005677 (leaving 58 variables) and λ=0.04698 (leaving 23 variables) in the models for the NMR metabolomic biomarkers and clinical biomarkers, respectively. LASSO, least absolute shrinkage and selection operator; NMR, nuclear magnetic resonance.

Supplementary Figure 7. Associations between metabolic biomarkers and FI in the UK Biobank, stratified by age groups, sex, and in non-white ethnic groups

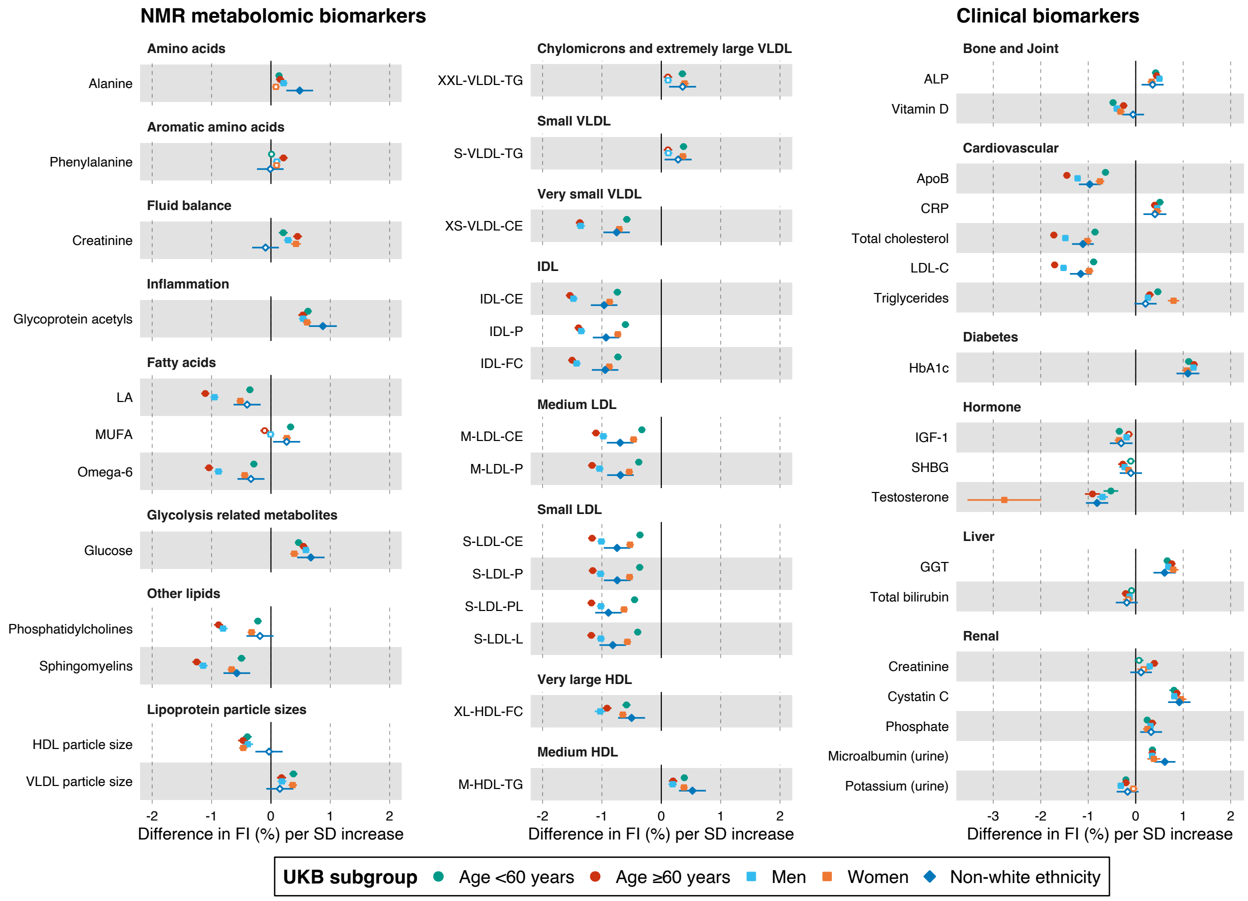

Notes: The estimates are from linear regression models adjusted for age, sex, baseline assessment center (England, Wales, Scotland), body mass index (continuous), smoking (never, previous, current), alcohol consumption (less than 3 times a month, 1–4 times a week, daily or almost daily), education level (high, intermediate, low), and deprivation index. The effect sizes represent changes in FI (%) per 1 standard deviation increase in biomarker level. Filled symbols represent statistically significant associations at $p<.00025$ (Bonferroni-corrected p-value threshold). Estimates for the subgroup analyses are shown in **Supplementary Tables 8–9**. ALP, alkaline phosphatase; ApoB, apolipoprotein B; C, cholesterol; CE, cholesteryl esters; FC, free cholesterol; FI, frailty index; GGT, gamma glutamyltransferase; HbA1c, glycated hemoglobin; HDL, high-density lipoproteins; IDL, intermediate-density lipoproteins; IGF, insulin-like growth factor; L, total lipids; LA, linoleic acid; LDL, low-density lipoproteins; M, medium; MUFA, monounsaturated fatty acids; NMR, nuclear magnetic resonance; P, particle concentrations; PL, phospholipids; PUFA, polyunsaturated fatty acids; S, small; SD, standard deviation; SHBG, sex hormone binding globulin; TG, triglycerides; Unsaturation, degree of unsaturation; UKB, UK Biobank; VLDL, very low-density lipoproteins; XL, very large; XS, very small; XXL, extremely large.
